# Results after intraoperative open and endovascular revascularization of acute mesenteric ischemia requiring a laparotomy

**DOI:** 10.1007/s00423-023-03035-8

**Published:** 2023-08-10

**Authors:** Marvin Kapalla, Rahul Choubey, Jürgen Weitz, Christian Reeps, Steffen Wolk

**Affiliations:** Department of Visceral, Thoracic and Vascular Surgery, University Hospital Carl Gustav Carus, Dresden, TU Germany

**Keywords:** Acute mesenteric ischemia, Endovascular treatment mesenteric ischemia, Surgical treatment mesenteric ischemia, Retrograde open mesenteric stenting

## Abstract

**Background:**

Acute mesenteric ischemia (AMI) is a dreaded condition with a difficult diagnosis and high mortality. Due to different baseline situations, the frequently performed comparison between endovascular and open surgical treatment is interfered with selection bias. The purpose of this study was to review outcomes in AMI treatment with an open or endovascular approach in association with laparotomy and to evaluate the endovascular-first strategy in similar clinical situations.

**Methods:**

The clinical data of 74 patients treated for AMI from 2007 to 2021 were retrospectively reviewed and compared. In-hospital mortality was appointed as the primary study endpoint. Risk factors for mortality were identified by using univariate and multivariate analysis.

**Results:**

In total, 61 patients (82%) were treated open surgically (OT) and, 13 patients (18%) with an endovascular approach (ET) in combination with laparatomy. The etiology of AMI was 49% arteriosclerotic and 51% thromboembolic occlusions. The total in-hospital mortality manifested at 43% (*n *=32) (OT 41% vs. ET 53.8%; *P*=0.54). As independent risk factors for in-hospital mortality, pneumatosis intestinalis (*P*=0.01), increased lactate concentration (*P*=0.04), and ischemic intestinal sections (*P*=0.01) were identified. Additionally, on univariate analysis patient age, congestive heart failure (> NYHA II) and atrial fibrillation were related with higher mortality.

**Conclusions:**

Morbidity and mortality of AMI remains at a high level. Conventional open or intraoperative endovascular therapy achieved similar results in patients with indication for laparotomy. Advanced disease stage with ischemic intestinal sections at presentation and cardiovascular comorbidities were associated with adverse outcome.

## Introduction

Acute mesenteric ischemia (AMI) occurs because of a sudden reduction in intestinal perfusion due to occlusive or non-occlusive arterial or venous vessel obstruction [1]. When untreated, AMI causes irreversible bowel necrosis with bacterial translocation, sepsis, and death [2]. The estimated incidence of AMI in patients with acute abdomen is around 1% and increases with age exponentially, up to 10% in patients over 70 years [3, 4]. Acosta et al. determined in an autopsy study an overall incidence rate of 12.9/100.000 people annually, hence the incidence rate based on hospital admissions may underestimate the prevalence of AMI [4, 5]. Concomitant with the improving life expectancy of the population and the associated increasing incidence of cardiovascular diseases, the risk for the development of mesenteric ischemia will also rise [2, 3]. Unfortunately, regardless of continuous improvement in vascular surgical skills and technical advancement in diagnostic imaging in recent decades, the mortality rate of AMI remains still high at 40–50% [3, 6–9]. Especially in the early stages, when the treatment would have been most beneficial [2], the unspecific symptoms and insufficient differential diagnostic involvement, cause a fatal delay in treatment. Since the establishment of endovascular procedures for the revascularization of visceral vessels in patients with chronic mesenteric ischemia, the endovascular approach is also increasingly preferred in AMI patients [10]. Compared to standard open surgery, the endovascular approach offers some theoretic advantages, such as a potentially faster restoration of the bowel perfusion which may limit the ischemic insult and help avoid laparotomy [11]. To date, there are three surgical society guidelines with a tendency for an endovascular-first approach [2, 9, 12]. Nevertheless, most comparative series are limited by small case numbers or short-term follow-up, so the quality of the supporting data is still restricted and the effect on the outcomes remains unclear. Additionally, most patients are already in critical condition at diagnosis or in a too advanced stage of disease for primary initial endovascular treatment, leading to a discrepancy between the theory in the literature and actual clinical practice.

The purpose of this study was to review outcomes in patients treated for AMI in similar clinical conditions with an open or endovascular approach in association with laparotomy and to evaluate the endovascular-first strategy in clinical practice.

## Methods

### Data collection and study population

**Fig. 1:**
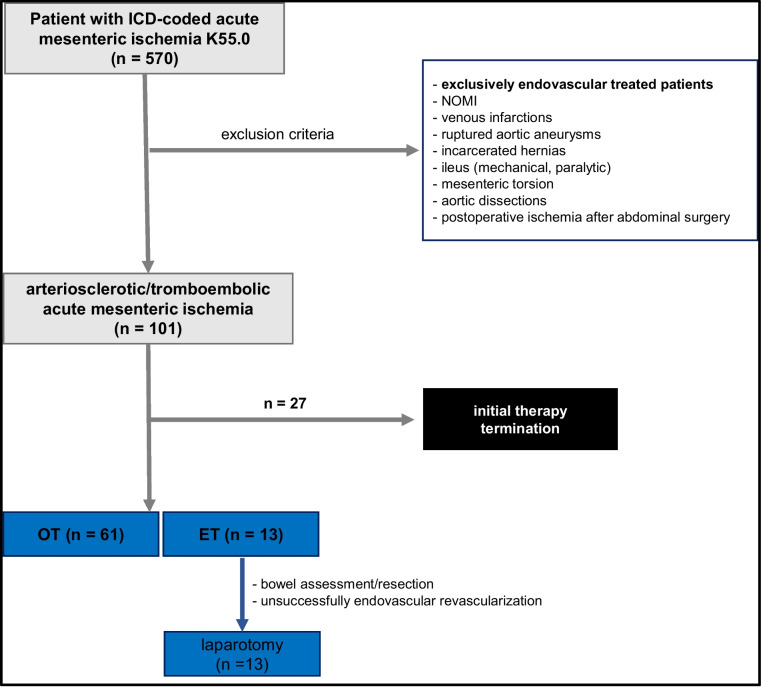
Therapy flowchart

All cases with an acute mesenteric ischemia treated with an open surgical or an endovascular approach between 01/2007 and 01/2021 were recorded at the Department of Visceral, Thoracic and Vascular Surgery of the Carl Gustav Carus University Hospital, Dresden. Patients were identified by their ICD coding (K55.0) via a query of the hospital's own electronic patient database. The data of each case were analyzed retrospectively. Inclusion criteria were patients diagnosed with AMI through clinical symptoms (acute abdominal pain, acute abdomen) and evidence of thromboembolic or arteriosclerotic occlusions of at least one mesenteric main stem vessel by computed tomography angiography (CTA). Differentiation from chronic mesenteric ischemia was based on clinical presentation (sudden symptom onset, no history of angina abdominalis). Patients who underwent an endovascular procedure were included only if they had an intraoperative endovascular revascularization in association with laparotomy for assessment of bowel viability or bowel resection (Fig. 1). Patients who underwent endovascular treatment exclusively by interventional radiology were excluded. Further exclusion criteria were patients with AMI due to rupture of an aortic aneurysm, aortic dissection, non-occlusive mesenteric ischemia (NOMI), venous occlusion, incarcerated hernia, ileus, mesenteric torsion or intestinal anastomotic insufficiency after visceral surgery as well as incomplete records. Furthermore, demographics, comorbidities, clinical presentation, radiologic data (calcification, occlusion localization, occlusion length), treatment modalities, complications and, follow-up examinations were collected.

### Ethical approval

All procedures performed in studies involving human participants were in accordance with the ethical standards of the institutional research committee and with the 1964 Helsinki declaration and its later amendments or comparable ethical standards. Per the guidelines for research on human subjects, approval was obtained from the local ethics committee at the Technische Universität Dresden (decision number EK 26012018). The ethics committee is registered as an institutional review board (IRB) at the Office for Human Research Protections (OHRP) (registration number IRB00001473 and IORG0001076).

### Indications and surgical/interventional technique

**Fig. 2:**
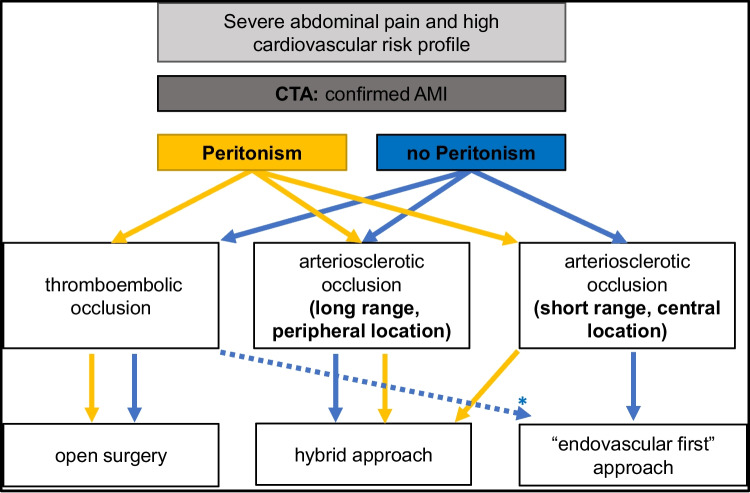
AMI treatment algorithm

Indication for revascularization was set in the presence of clinical symptoms of AMI, CTA confirmation of acute visceral vessel occlusion and after exclusion of other differential diagnoses. In our center, an interdisciplinary therapy approach consisting of vascular surgery, visceral surgery and around-the-clock available interventional radiology, has been established (Fig. 2). First, depending on the clinical situation, it is decided whether the patient requires primary open surgical treatment (hemodynamic instability, peritonism, evidence of manifest intestinal ischemia on CT). Furthermore, a revascularization procedure is selected depending on the type of occlusion and its extent. Thromboembolic occlusions are primarily treated by open surgery, whereas arteriosclerotic occlusions with short and centrally located occlusions primarily by endovascular treatment.

Open treatment included embolectomy, arterial bypass or thrombendarterectomy and if necessary, bowel resection. Access was created via a midline laparotomy. All patients received perioperative antibiotic prophylaxis with ceftriaxone and metronidazole. Depending on the pathology and anatomic conditions a retrograde (iliaco-mesenteric, iliaco-celiac) or antegrade (aorto-celiac + aorto-mesenteric) bypass was established. In cases of thrombendarterectomy, a venous patch reconstruction was performed. In unclear ischemic intestinal damage after revascularization, a second-look laparotomy was routinely indicated. All open or hybrid procedures were performed in a hybrid operating room.

Endovascular treatment included an endovascular approach for revascularisation with subsequent open surgical treatment for evaluation of ischemia and, if necessary, intestinal resection as well as retrograde open mesenteric stenting. Percutaneous transfemoral or transbrachial access was used depending on the angulation between the visceral arteries and the aorta. During open and endovascular approaches, systemic heparin was administered to maintain activated clotting times greater than 250 s. The activated clotting time was checked in 20-min intervals. After a 5 French sheath was introduced using the Seldinger technique into the femoral or brachial artery, the abdominal aorta was catheterized to perform an aortography to localize the ostium of the target vessel. A long 5- to 8- French sheath (depending on the type and diameter of the stent) was introduced into the celiac trunk or the superior mesenteric artery and a 0.035” stiff guidewire was placed into a peripheral branch of the artery. After the determination of the adequate diameter and length in the peri-interventional angiography the stent was placed into the vessel and released. Balloon-mounted or self-expanding bare-metal stents were used (Express Vascular SD, Boston Scientific; Marlborough, MA, USA and Astron Pulsar ®; BIOTRONIK SE & Co. KG, Germany). In cases of retrograde open mesenteric stenting (ROMS) after a midline laparotomy, the superior mesenteric artery (SMA) is exposed and a flexible 7 French sheath was introduced in a retrograde fashion using the Seldinger technique and the outflow was occluded through vascular clamps. After crossing the lesion with a 0.035-inch stiff guidewire a stent angioplasty was performed in the way described above. In case of (heavily) calcified lesions, predilatation was performed. In case of longer occlusions, the stent was elongated with a self-expandable stent. Before the sheath removal, completion angiography was routinely performed in two projections. Technical success was defined with the return of bowel perfusion without evidence of a residual stenosis in the final angiography.

All patients received antiplatelet therapy after the procedure. After stent implantations, patients received dual antiplatelet therapy with acetylsalicylic acid (ASA) 100 mg and clopidogrel 75 mg for 6 weeks, then Aspirin 100 mg daily as lifelong monotherapy. Routine follow-up consisted of clinical examination and duplex sonography every 3 and 6 months during the first year and at least annually hereafter.

### Outcome parameters and definitions

The primary endpoint of this study was in-hospital mortality. As secondary outcomes technical success, morbidity, long term survival and revision rates were evaluated.

Since the time from symptom onset to treatment could not often be exactly recorded, the time between primary admission and the commencement of treatment (surgical incision), was documented. The etiology was determined as (thrombo-)embolic and thrombotic on arteriosclerotic occlusions based on the operation and radiological findings. Stenosis ≥ 70% was considered to be high-grade. Technical success was defined a restoration of intestinal perfusion and in the case of arteriosclerotic lesions 30 % residual stenosis by angiography or intraoperative duplex sonography (peak systolic velocity of less than a threshold of 3 m/s and without post-stenosis signal).

All reinterventions/reoperations after the primary successfully treatment were denoted as vascular revisions. Complications were categorized according to the Clavien-Dindo classification and recorded when they exceeded grade I [13]. Early postprocedure period was defined as the first 30 days after treatment or with in-hospital stay if these were more than 30 days. Follow-up period was defined as discharge from the hospital until the last available clinical examination. Risk factors for endovascular failure were identified using uni- and multivariate analysis.

### Statistical analysis

The statistical analysis was performed using IBM SPSS for Windows, version 21.0 (IBM Corp., Armonk, NY). All clinical characteristics were grouped to build categorical or nominal variables. Dichotomous variables were recorded as absolute frequencies (number of cases) and relative frequencies (percentages). Continuous data were presented as mean and standard deviations for parametric data or median values and interquartile ranges for nonparametric data. Pearson’s chi-squared or Fisher exact test was used for the analysis of categorical variables. Differences between means were tested with a *t*-test or Mann-Whitney-*U*-test. A binary logistic regression analysis was performed to model the influence of the variables on perioperative mortality, which were significant in the univariate analysis. The odds ratio (OR) as an estimate of relative risk between two groups based on the mortality was specified with their 95% confidence interval (CI 95%). Survival and patency data was analyzed using Kaplan-Meier estimates and differences were appointed by the log-rank test. A two-sided *P*-value < 0.05 was considered statistically significant.

## Results

### Study population and patient characteristics

Seventy-six patients (male *n* = 53, 71.6%; female *n* = 21, 28.4%) with a mean age of 73.6 ± 11.7 years were included. The therapy of AMI was open treatment (OT) in 61 patients (82.4%) and endovascular treatment (ET) in 13 (17.6%). A total of 48.6% of the patients had atrial fibrillation, which was previously unknown in 11% and untreated (no antiplatelet or anticoagulation therapy) in 12.8% of the cases. Other common comorbidities and risk factors were hypertension (87,8%), chronic kidney disease [GFR < 30ml/min/1.73m^2^] (29.7%), diabetes mellitus (43.2%) and congestive heart failure [NYHA II] (23%) (Table 1).

**Table 1:** Demographic and clinical data

Variables*	*n = 74 (%)*
*Demographic data*	
Age (years)	73.6 ± 11.7
Sex (male/female)	53/21 (72/28)
*Risk factors and comorbidities*	
BMI (kg/cm^2^)	24.3 ± 5.1
Chronic kidney disease**	22 (31)
Congestive heart failure (>NYHA II)	17 (23)
Atrial fibrillation	36 (49)
Hypertension	65 (88)
CHD	22 (30)
PAOD (> Fontaine stage II)	25 (34)
Hyperlipidemia	26 (35)
Diabetes mellitus	32 (43)
COPD	10 (14)
Nicotine abuse	16 (22)
Alcohol abuse	4 (5)
Previous vascular surgery	20 (27)
*Diagnostics*	
Pneumatosis intestinalis	17 (23)
Free intraperitoneal air	6 (8)
Intestinal wall thickenings	36 (49)
LDH [> 4,2U/I]	35 (65)
Lactate [> 2,2mmol/l]	44 (60)
pH [< 7,35]	28 (38)
Leukocytes [ > 11.300/μl]	56 (76)

*BMI* body mass index, *CHD* coronary heart disease, *COPD* chronic obstructive pulmonary disease, *PAOD* peripheral arterial occlusive disease, *LDH* lactate dehydrogenase

*Continuous data presented as mean ± standard deviation

**GFR < 30 ml/min/1.73m^2^

### Clinical presentation and treatment of AMI

In the clinical examination, abdominal pain was the most common symptom in all the patients, followed by signs of peritonism (31.1%), vomiting (25.7%) and diarrhea (16.2%). Bloody diarrhea was documented in 13.2% of the cases, further 13.2% presented with tachycardia and hypotension (shock). Auscultatory 18.4% showed no abdominal sounds, whereas 2.6% had high pitched sounds. At the time of admission, 10.5% (*n* = 8) of the patients were intubated, of whom 7.9% (*n* = 6) were transferred from peripheral hospitals. Initial clinical diagnosis of the AMI was correctly placed only in 62.1% (*n *=46) of the patients.

A total of 108 vessels were occluded. Forty-four patients (59.5%) had one vessel, 26 patients (35.1%) had two vessels, and four patients (5.4%) all three vessels occluded. Participation of the superior mesenteric artery (SMA) was significantly more frequently found in all (100%) patients as participation of the celiac trunk (CT) in 39.2% or of the inferior mesenteric artery (IMA) in 8.1% (Table 2). The etiology was acute atherosclerotic occlusion in 48.6% and thrombo-embolic in 51.4% of the patients. Occlusion localization of the SMA at the origin was seen in 51.4% (*n* = 38), whereas central or peripheral occlusion (> 5cm from vessel origin) was present in 32.4% (*n* = 24) and 16.2% (*n* = 12), respectively. Long-range SMA occlusion was more frequent (64.9%, *n* = 51) than short-range (31.1%, *n* = 26). Further radiographic findings were intestinal wall thickenings in 36 patients (48.6%), pneumatosis intestinalis in 17 (23%) and free intraperitoneal air in six patients (8.1%). In the setting of embolism, a cardiac source of embolism was confirmed in 67% of cases. Frequent laboratory findings included elevated lactate, LDH and CRP concentrations as well as degraded blood pH values and leukocytosis (Table 1).

**Table 2:** Vessels

Variables	*n = 74 (%)*
*Extension of disease*	
Single-vessel disease	44 (60)
Two-vessel disease	26 (35)
Three-vessel disease	4 (5)
∑	108
CT	29 (39)
SMA	74 (100)
IMA	6 (8)
*Etiology*	
Arteriosclerotic occlusion	36 (49)
Thrombo-embolic	38 (51)
*SMA occlusion localization*	
Vessel origin	38 (51)
Central	24 (32)
Peripheral (> 5cm)	12 (16)
*SMA occlusion lengths*	
Short-range (< 2cm)	26 (35)
Long-range (> 2cm)	48 (65)
*Vessel revascularization*	
Single-vessel revascularization	64 (86)
Two-vessel revascularization	10 (14)
Three-vessel revascularization	-
∑	84

*SMA* superior mesenteric artery, *CT* coeliac trunk, *IMA* inferior mesenteric artery

In ET (*n *=13), antegrade stent angioplasty was performed in 12 patients (92.3%) and ROMS in one patient (7.7%). Five endovascular interventions (38.5%) were unsuccessful, as the mesenteric vessel occlusion could not be passed or entered. These patients received immediate open revascularization. Further analysis of these patients was performed as intention-to-treat (ITT). Only arteriosclerotic occlusions were treated endovascularly (100%, *P* < 0.001). The occlusion length was shorter (2.07cm ± 0.63cm vs. 3.89cm ± 1.98cm; *P* = 0.01), and the occlusion localization was more centrally located compared to the primary open surgical approach (0.72cm ± 0.88cm vs. 2.41cm ± 2.79cm; *P* = 0.09).

In OT (*n *=61), method of revascularization was embolectomy or thrombectomy in 35 patients (57.3%), bypass implantation in 17 (27.8%) and thrombendarteriectomy with venous patch in nine (14.7%). The type of bypass in initial surgery was retrograde in 16 patients (iliaco-mesenteric *n *=13; iliaco-mesenteric + iliaco-coeliac *n* = 3) and antegrade in one case (aorto-mesenteric). Silver grafts were used in eight patients (47.1%), autologous vein (V. saphena magna) in *n* = 2 (11.8%), PTFE grafts *n* = 3 (17.6%) and Dacron in *n* = 4 (23.5%). All thromboembolic occlusions were treated primarily by open surgery.

A second-look laparotomy was performed in 79.7% (*n *=59) of the patients. Initial bowel resection was necessary in 70.3% (*n *=53) of the cases. In case of resection, isolated Ileum segment resection was most commonly performed (39.6%, *n *=21), followed by combined small and large bowel resection (28.3%, *n *=15) and right hemicolectomy (26.4%, *n *=14). A total of 84 vessels were treated, single vessel revascularization in 64 patients (86.5%) and two vessels revascularization in ten patients (13.5%), a three vessels revascularization was not performed.

### Patient outcome

According to the Clavien-Dindo classification, 85.1% (*n *=63) patients developed complications greater than grade IIIa (Table 3) [13]. The most common complications were acute kidney injury (44.6%), sepsis (56.8%), pulmonary complications (28.4%), and wound healing disorders (14.9%). Furthermore, in the postoperative course 22 patients (29.7%) which have already undergone an initial resection, required additional resection from remaining ischemic intestinal sections.

**Table 3:** Postoperative course and outcomes

Variable*	*n = 74 (%)*
*Complications*	
Additional resection**	25 (34)
Wound healing disorder	11 (15)
Short-bowel syndrome	8 (11)
Aneurysm spurium	2 (3)
Acute kidney injury	36 (45)
Cerebrovascular complications	14 (19)
Cardiac complications	12 (16)
Pulmonary complications	21 (28)
Sepsis	42 (57)
Septic multiorgan failure	26 (35)
*Clavien-Dindo classification*	
grade II	11 (15)
grade IIIa	4 (5)
grade IIIb	14 (19)
grade IVa	8 (10)
grade IVb	5 (7)
grade V	32 (43)
*Outcomes*	
Perioperative mortality	32 (43.2)
Hospital length of stay, median (range)	23 (11 - 42)
Intensive care unit stay, median (range)	13 (5 - 29)

*Continuous data presented as mean ± standard deviation or median and range

**From initially remaining ischemic intestinal sections

The in-hospital mortality of all revascularized patients (*n *=74) was 43.3% (*n* = 32) without a significant difference in treatment modalities (OT 41% [*n *=25] vs. ET 53.8% [*n *=7]; *P* =0.54) or a significant decrease during the last 10 years. For the entire cohort, hospital stay was 23 days (range 14–37 days) and intensive care unit stay was 11 days (range 5–26 days).

A total of four patients (5.4%) needed vascular reoperations or reinterventions. In the early postprocedural period two OT patients (3.3%) required vascular revision due to complications (bleeding complication *n* = 1 and re-thrombosis *n* = 1).

**Fig. 3:**
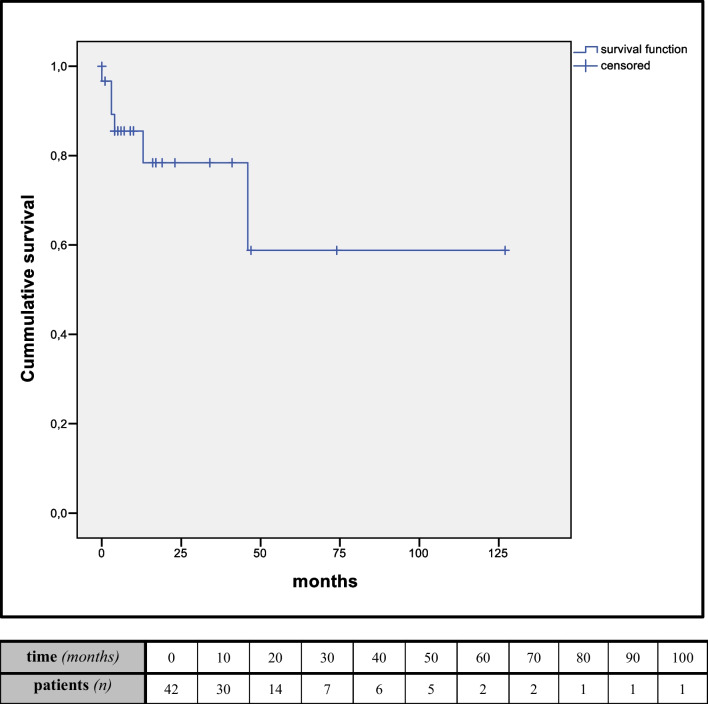
Kaplan-Meier estimates of overall survival

The mean follow-up was 19 months (range 6.5–46 months) and available in 30/42 patients (65.3%). There were seven late deaths. Kaplan-Meier estimates for 1-year and 5-year survival were 85.5% and 58.8% (Fig. 3)*.* The cause of death was malignancy in three, myocardial infarction in two, and unknown in two patients.

Only further two OT patients received endovascular stent implantation during follow-up into the inserted bypass. One patient required the reintervention after three months due to kinking stenosis and another patient after 28 months due to re-stenosis in the anastomotic region.

### Risk factors for poor survival

Univariate analysis identified age (77.1 ± 10.1 years vs. 71 ± 12.2 years; *P* = 0.03), increased BMI (27.5 ± 6.6kg/cm^2^ vs. 24.3 ± 6.1kg/cm^2^; *P* = 0.04), atrial fibrillation (69% vs. 31%; *P* = 0.03), increased lactate (*P* = 0.04) and decreased blood pH values (*P* = 0.03) as negative predictors for survival (Table 4). Patients with an initial wrong clinical diagnosis tended to survive worse 37.5% (*n *=12) vs. 62.5% (*n *=20) (*P =* 0.07). Further on, patients with pneumatosis intestinalis (71% vs. 29%; *P* = 0.01) as well as patients with remaining ischemic intestinal sections (60% vs. 40%; *P* = 0.02), postoperative complications such as acute kidney injury (61% vs. 39%; *P* < 0.01), septic multiorgan failure (92% vs. 8%; *P* < 0.01) and longer intensive care unit stays (*P* < 0.01) showed a poorer survival*.*

**Table 4:** Factors associated with in-hospital mortality in *n* = 74 patients

Variable*	Deceased *n* = 32	Survived *n* = 42	*P*
	*n (%)*	*n (%)*	
Age (years)	77.1 ± 10.1	71 ± 12.2	.03
BMI [kg/cm^2^]	27.5 ± 6.6	24.3 ± 6.1	.04
Congestive heart failure (> NYHA II)	13 (62)	8 (38)	.04
Atrial fibrillation	25 (69)	11 (31)	.03
wrong referral diagnosis	15 (54)	13 (46)	0.54
Etiology			.11
arteriosclerotic	19 (53)	17 (47)	
thromboembolic	13 (34)	25 (66)	
SMA origin occlusion	21 (44)	17 (59)	.05
Vessel involvement			.82
One vessel	19 (43)	25 (57)	
Two vessels	11 (42)	15 (58)	
Three vessels	2 (50)	2 (50)	
Pneumatosis intestinalis	12 (71)	5 (29)	.01
Lactate [> 2.2 mmol/l]	25 (56)	20 (44)	.04
pH Value [< 7.35]	17 (61)	11 (39)	.03
Acute kidney injury	20 (61)	13 (39)	<.01
Ischemic intestinal sections	18 (60)	12 (40)	.02
Septic multiorgan failure	24 (92)	2 (8)	<.01
Intensive care unit stay, median (range)	17 (8 - 31)	11 (3 - 24)	.02

*Continuous data presented as mean ± standard deviation or median and range

Patients with a thromboembolic nature of the occlusion tended to survive better (thromboembolic 59.5% vs. 40.6% and arteriosclerotic 40.5% vs. 59.4%), but at a non-significant level (*P* = 0.11)*.* Also, non-significant higher mortalities were observed in patients with multiple vessel occlusions (one vessel 43% vs. two vessels 42% vs. three vessels 50%; *P = 0.82*). Initial endovascular failure had no significant influence on survival (66.7% vs. 33.3%; *P =* 0.59).

In multivariate analysis, predictors of mortality were raised lactate concentration (OR 1.5; 95% CI 1.1–13; *P* = 0.04), pneumatosis intestinalis in CTA (OR 1.4; 95% CI 1.1–6.7; *P* = 0.01) and remaining ischemic intestinal sections in second-look operations (OR 3.5; 95% CI 1.3–13.9; *P* = 0.01) (Table 5)*.*

**Table 5:** Multivariate analysis of predictors associated with in-hospital mortality in *n = 74* patients

Variable*	*OR*	*95 % CI*	*P*
Lactate [mmol/l]	1.5	1.1–13	.04
Pneumatosis intestinalis	1.4	1.1–6.7	.01
Ischemic intestinal sections	3.5	1.3–13.9	.01

*CI* confidence interval,*OR* odds ratio

## Discussion

The major issue in the treatment of AMI is the delay in diagnosis and treatment due to unspecific abdominal symptoms. This is also underlined by our own results with a strong tendency to worse outcome, if admission diagnosis of AMI was delayed. Therefore, the European Society for Trauma and Emergency Surgery (ESTES) recommends, that “AMI should be suspected in patients with acute abdominal pain in whom there is no clear diagnosis, particularly when the pain is disproportionate to the physical examination findings and in the elderly with a history of cardiovascular comorbidities” [2]. Consistent with our results, Schermerhorn et al. evaluated in the so far largest data analyses from the Nationwide Inpatient Sample (NIS), that mainly elderly patients with high comorbidities of cardiovascular diseases were affected by AMI and identified also age and atrial fibrillation as a predictor of poor survival [14]. Furthermore, we observe that congestive heart failure or increased BMI had a negative influence on patient survival. Clinically, most patients presented with unspecific symptom complex of abdominal pain (100%), peritonism (31%), vomiting (26%) and diarrhea (16%), but also 11% of the patients were already mechanically ventilated at the time of presentation. Therefore, a symptom-oriented diagnosis was impeded. In a review, Eckstein et al. demonstrated that above a time interval from 12 – 24 hours between symptom onset and treatment, an exponential increase in mortality up to 70–95% can be expected [15]. Due to our retrospective study design, we were not able to record the time between symptom-onset and treatment begin reliably. Additionally, a time registration between primary admission and treatment begin seems pointless as results would be biased by unrecorded prehospital events. Primary referral diagnosis of AMI was placed correctly in only 62% of the patients. Even so, patients with a wrong referral diagnosis had worse survival than patients with a correct referral diagnosis (38% vs. 63%; *P*= 0.07).

In older published studies, thromboembolic occlusions were the most common etiology with 35–45% [15, 16]. In our study, these patients (51%) showed higher rates of atrial fibrillation (71%; *P* < 0.01) and a more fulminant clinical symptomatology. As with other authors in recent years, an increase in arteriosclerotic occlusions to the main etiology was also observed in our study (49%) [11, 17]. As reasons for this Eckstein et al. mentioned the increased use of anticoagulants and the decreasing incidence of rheumatic valvular diseases as well as the higher incidence of degenerative vascular diseases in the rising average age of the population [15]. Moreover, in our study patients with thrombotic occlusions had significantly higher rates of POAD (76%; *P* < 0.01). Moreover, arteriosclerotic occlusions were associated with higher mortality compared to embolic occlusions (53% vs. 34%; *P* = 0.11), probably due to a larger and more poorly compensated bowel ischemia [1, 11].

The CTA was the imaging modality of choice in all cases and has proven to be highly sensitive and specific for the diagnosis of AMI [4, 18]. In accordance with the literature, the SMA was occluded in all patients [15, 17]. According to Bala et al. the SMA is predestined for embolic events due to the large vessel diameter and sharp angulation [19]. Further findings in preoperative diagnostic associated with mortality were pneumatosis intestinalis, increased lactate and LDH concentration as well as decreased blood pH-values (Tables 4 and 5). Overall and as already confirmed in many studies, the existing laboratory diagnostic for AMI is only rarely specific and therefore only to be regarded as additive [20]. The further clinical role of new plasma markers such as the “intestinal fatty acid-binding protein” (I-FABP) or the α-glutathione S-transferase [21] remains to be seen, the validity still has to be investigated in further studies [4, 22].

Whether to treat AMI by primary open or endovascular approach remains a major discussion. To date, there are three surgical society guidelines with a tendency for an endovascular-first strategy [2, 9, 12]. Also, in our center, an endovascular approach for AMI treatment was used more frequently in recent years. But in our practical experience, most patients are already in critical condition at diagnosis or in a too advanced stage of disease for primary initial endovascular treatment as recommended. In the presence of peritoneal irritation and/or severe radiologic findings, an open approach is indicated and leads to appropriate patient selection for the endovascular procedure and to a discrepancy between the theory in the literature and actual clinical practice. Therefore, we focused our study exclusively on patients who received open or intraoperative endovascular surgery with subsequent laparotomy to limit the bias of the results. Endovascular approach was particularly used in patients with arteriosclerotic occlusion near the ostia. Endovascular aspiration thrombectomy, mechanical thrombectomy, or thrombolysis are rarely used in our clinic and only applied for selected cases. Especially in patients who require a laparotomy, an open thrombectomy can be performed directly. We consider the trauma caused by additional thrombectomy of thromboembolic occlusions to be low. The frequently performed comparison between endovascular and open surgical therapy without appropriate risk stratification of the patients does not seem reasonable to us, simply because different baseline situations are compared.

Furthermore, it must be mentioned that in 38.5% of the patients the endovascular approach was technically unsuccessful due to the impossibility of probing the target vessel. As reported in a recent meta-analysis by Hou et al. conversion rates for endovascular treatment are 40-72% [8]. In fact, only arteriosclerotic occlusions were treated by ET so that the endovascular failure can be explained by the heavily calcified visceral aortas and mesenteric vessel orifices [11, 23]. However, endovascular failure did not increase mortality rates (*P* = 0.59).

The literature about retrograde open mesenteric stenting (ROMS) is still limited [8]. A recent multicenter study showed that ROMS can be used in patients with AMI who do not respond to percutaneous endoluminal therapy due to mesenteric occlusion or are in need for laparotomy for exploration and treatment of ischemic bowel [24]. Theoretically, if medical institutions are equipped for hybrid surgeries and this treatment option was organizationally considered in advance, ROMS may be a good treatment option for patients who would require laparotomies anyway to minimize the time and extent of the operation and to eliminate the risk of surgical bypass graft infection.

Our findings in perioperative course associated with mortality are consistent with other published studies despite the fact that only patients in clinically advanced stages with indication for laparotomy were included [8–11, 14, 25, 26]. The high rate of postoperative acute renal failure (45%) is probably conditioned by the high prevalence of chronic renal insufficiency (35%) in our cohort. The in-hospital mortality for revascularized patients (*n* = 74) was 43.3%, without a significant difference in patients provided by OT (41%) or ET (53.8%) (*P* = 0.54). As mentioned above, we focused exclusively on surgically or endovascular treated patients with subsequent need for laparotomy and excluded purely endovascularly treated patients due to their better initial condition, which certainly worsens our mortality outcomes.

In conclusion, the heterogeneous study designs and lacking reporting standards for AMI lead to limited comparability of mortality in the treatment modalities. A randomized trial would be necessary but difficult to implement owing to the infrequent and acute manifestation of these diseases.

This study has some limitations. It is a retrospective single center study, generating bias linked to a retrospective data collection. Furthermore, during the long study period, there has been a progress in medical therapy and endovascular technology that may have affected patients’ outcomes. Given the small number of patients treated with ET and the limited follow up, we did not believe that a comprehensive comparison would be appropriate, and also due to the fact that a selection bias cannot be avoided.

## Conclusions

In conclusion, the mortality of AMI remains at a high level. Endovascular therapy can complement surgical revascularization for short central arteriosclerotic occlusions. However, these morphological constitutions represent a minority, so in actual practice open surgical revascularization plays a major role. The ideal method of revascularization should be a patient-specific concept in each case depending on the clinical situation and the morphology of the occlusion. As independent risk factors for in-hospital mortality pneumatosis intestinalis, increased lactate concentrations and ischemic intestinal sections were identified. Due to the initial high morbidity of the patients, fast pre- and in-hospital procedures are particularly necessary to minimize the time factor, which is next to an interdisciplinary approach in diagnostics and therapy decisive for success of the therapy and improvement of prognosis. Accordingly, the appropriate therapy should be selected on an individual approach depending on the patient's clinical condition.

## Data Availability

Not applicable.
